# Book Reviews

**Published:** 1913-06

**Authors:** 


### BOOK REVIEWS

THE MOSQUITO, ITS RELATION TO DISEASE AND ITS EXTERMINATION. By Alvah H. Doty, Formerly Health Officer of the Port of New York. Illustrated. 79 pages. Published by D. Appleton & Co., New York.

Doty has brought together in a plain, practical way, the information needed by those fighting the mosquito and the diseases carried by it. The book is so simply written that a layman could well understand it.

---

SOLIDIFIED CARBONDIOXIDE IN THE TREATMENT OF SKIN DISEASES. By Ralph Bernstien, M. D. Clinical Instructor in skin diseases, Hohnemann Medical College, Philadelphia. Published by Frank S. Betz Co., Hammond, Ind.

The writer believes that we have in solidified carbondioxide, the remedial substance per excellence in the treatment of cutaneous neoplasms, both benign and malignant. Many illustrations are given showing patients before and after treatment.

---

COMPLETE METHODICAL BIBLIOGRAPHY OF MEDICAL, PHARMACEUTICAL, CHIRURGICAL AND SCIENCE BOOKS. In Octo. 128 pages, with engravings. Free.

The New Edition of the Methodic Bibliography of Medical Books, entirely renovated, give, put up by chapters, the name with notice of all medical works published in France between 1900 and 1913.

A supplement gives the names of books published during 1912-13 and those in preparation.

This vadecum of bibliography is indispensable to all physicians. It is sent free, post-paid, upon application to

GRAND LIBRAIRIE MEDICAL

A MALOINE

25-27 rue de l' Ecole- de-Cedecine,

Paris, France.

W. B. SAUNDERS COMPANY, of Philadelphia and London, have issued another edition (17th) of their handsome illustrated catalogue.

In going through this edition we find it describes new books and ten new editions, not described in the previous issue. These new books are of great interest to the medical man, because they treat of subjects being daily discussed in medical circles.

Any physician can get a copy of the Saunders' catalogue by dropping a line to these publishers. A copy should have a place on the desk of every physician, because it is most valuable as a reference work of modern medical literature. Send to Saunders today for a copy.

---

W. B. Saunders Company, Publishers of Philadelphia and London, have issued another edition (17th) of their handsome illustrated catalogue.

In going through this edition we find it describes nine new books and ten new editions, not described in the previous issue. These new books are of great interest to the medical man, because they treat of subjects being daily discussed in medical circles.

Any physician can get a copy of the Saunder's catalogue by dropping a line to these publishers. A copy should have a place o nthe desk of every physician, because it is most valuable os a reference work of modern medical literature. Send to Saunders today for a copy.

---

THE MODERN HOSPITAL; ITS INSPIRATION; ITS ARCHITECTURE; ITS EQUIPMENT; ITS OPERATION. By John A. Honsby, M.D., Secretary Hospital Section, American Medical Association; Member American Hospital Association, etc., and Richard E. Schmidt, Architect, Fellow American Institute of Architects. Actavo volume of 644 pages with 207 illustrations. Philadelphia and London: W. B. Saunders Company, 1913. Cloth, $7.00 net: half morocco $8.50 net.

We would like to see this book carefully read by every Hospital superintendent. It is the most elaborate aand complete work in English upon this important subject. It affords the small hospital the opportunity to supply facilities almost equal to the large, fully equipped hospitals. Every important phase of the subject is adequately discussed. We highly commend the book.

---

THE SURGICAL CLINICS OF JOHN B. MURPHY, M.D., at Mercy Hospital, Chicago. Volume II Number II. (April 1913). Octavo of 171 pages, illustrated. Philadelphia and London: W. B. Saunders Company, 1913. Published Bi-Monthly. Price per year: Paper, $8.00. Cloth, $12.00.

This number of the Surgical Clinics of John B. Murphy contains the usual lucid condensed descriptions of a varied number of important surgical procedures. Gastrical and duodenal ulcers are discussed by Mr. Robert Milne, T. R. C. S. of London.

